# Supplementary material for: Comparison of 7 surgical interventions for recurrent lumbar disc herniation: A network meta-analysis and systematic review
Source: PLoS One. 2025 Mar 4;20(3):e0309343. doi: 10.1371/journal.pone.0309343 (PMC11878942; doi:10.1371/journal.pone.0309343)
Supplement: S1 Table — (DOCX) [file pone.0309343.s002.docx]

| Comparison | Direct | Indirect | Network | P.value |
| --- | --- | --- | --- | --- |
| MED.PELD | -0.60 (-2.4, 1.2) | -0.55 (-2.4, 1.3) | -0.35 (-1.7, 1.0) | 0.97 |
| MIS-TLIF.PELD | 0.62 (-0.37, 1.6) | 0.24 (-2.3, 2.8) | 0.64 (-0.21, 1.5) | 0.76 |
| MIS-TLIF.TLIF | 0.075 (-1.8, 2.) | 0.53 (-1.3, 2.3) | 0.31 (-0.97, 1.6) | 0.72 |
| OD.PELD | -0.087 (-0.95, 0.79) | 0.36 (-2.1, 2.8) | -0.042 (-0.82, 0.74) | 0.72 |
| OD.TLIF | -0.25 (-1.8, 1.3) | -0.69 (-3.2, 1.9) | -0.37 (-1.5, 0.74) | 0.74 |
| PLIF.TLIF | -0.23 (-1.8, 1.3) | -0.60 (-3.9, 2.7) | -0.29 (-1.5, 0.91) | 0.82 |

Table 1. Node-splitting analyses of VAS(back pain)

MED=Microendoscopic Discectomy.MIS-TLIF=Minimally Invasive transforaminal lumbar interbody fusion. OD= Open discectomy.PELD=Percutaneous Endoscopic Lumbar Diskectomy. PLIF= posterior lumbar interbody fusion.TLIF= transforaminal lumbar interbody fusion.Unilat TLIF= Unilateral transforaminal lumbar interbody fusion. VAS =visual analogue scale.

Table 2. Node-splitting analyses of VAS(leg pain)

MED=Microendoscopic Discectomy.PELD=Percutaneous Endoscopic Lumbar Diskectomy. VAS =visual analogue scale.

| Comparison | Direct | Indirect | Network | P.value |
| --- | --- | --- | --- | --- |
| MED.PELD | -0.48 (-2., 0.97) | -2.2 (-3.8, -0.63) | -1.1 (-2.9, 0.80) | 0.08 |

Table 3. Node-splitting analyses of ODI

MED=Microendoscopic Discectomy.MIS-TLIF=Minimally Invasive transforaminal lumbar interbody fusion. OD= Open discectomy.PELD=Percutaneous Endoscopic Lumbar Diskectomy. PLIF= posterior lumbar interbody fusion.TLIF= transforaminal lumbar interbody fusion.Unilat TLIF= Unilateral transforaminal lumbar interbody fusion.

ODI= Oswestry disability index.

| Comparison | Direct | |  | Indirect | |  | Difference | | P.value |
| --- | --- | --- | --- | --- | --- | --- | --- | --- | --- |
|  | Coef | Std. Err |  | Coef | Std. Err |  | Coef | Std. Err |  |
| PELD.MED | -2.38 | 1.84 |  | -0.72 | 2.66 |  | -1.66 | 2.82 | 0.56 |
| PELD.OD | -1.92 | 0.73 |  | -0.13 | 57.74 |  | -1.79 | 57.75 | 0.98 |
| MED.MIS-TLIF | 1.77 | 1.78 |  | 3.43 | 2.80 |  | -1.66 | 2.82 | 0.56 |
| TLIF.PLIF | 1.53 | 1.35 |  | 7.39 | 4.21 |  | -5.86 | 4.35 | 0.18 |
| TLIF.OD | 1.12 | 1.42 |  | -4.74 | 4.14 |  | 5.86 | 4.35 | 0.18 |
| PLIF.OD | -1.46 | 1.04 |  | -5.03 | 115.49 |  | 3.58 | 115.50 | 0.98 |

| Comparison | Direct | Indirect | Network | P.value |
| --- | --- | --- | --- | --- |
| PELD.MED | -0.52 (-2.8, 1.6) | 2.3 (0.26, 4.4) | 0.89 (-0.59, 2.3) | 0.06 |
| MED.MIS-TLIF | -1.3 (-5.1, 1.5) | -3.2 (-5.4, -1.1) | -1.9 (-3.4, -0.39) | 0.34 |
| MED.PLIF | -1.6 (-3.2, -0.15) | 1.1 (-1.3, 3.6) | -0.84 (-2.1, 0.51) | 0.05 |
| PELD.MIS-TLIF | -0.97 (-1.9, -0.087) | -1.0 (-3.8, 1.5) | -1.0 (-2., -0.13) | 0.96 |
| MIS-TLIF.TLIF | 0.29 (-1.5, 2.1) | -0.60 (-2.6, 1.5) | -0.10 (-1.5, 1.3) | 0.49 |
| TLIF.PLIF | 1.4 (-0.033, 2.9) | 0.48 (-2.2, 3.4) | 1.1 (0.026, 2.5) | 0.55 |
| PELD.OD | 0.49 (-0.72, 1.8) | -0.55 (-2.6, 1.5) | 0.22 (-0.82, 1.3) | 0.37 |
| TLIF.OD | 1.5 (-0.39, 3.8) | 1.3 (-0.56, 3.2) | 1.3 (0.095, 2.7) | 0.86 |
| PLIF.OD | -0.14 (-1.4, 1.1) | 1.2 (-1.2, 3.6) | 0.17 (-0.82, 1.2) | 0.32 |

Table 4 . Node-splitting analyses of complication

MED=Microendoscopic Discectomy.MIS-TLIF=Minimally Invasive transforaminal lumbar interbody fusion. OD= Open discectomy.PELD=Percutaneous Endoscopic Lumbar Diskectomy. PLIF= posterior lumbar interbody fusion.TLIF= transforaminal lumbar interbody fusion.Unilat TLIF= Unilateral transforaminal lumbar interbody fusion.

Table 5 . Node-splitting analyses of recurrence rate

MED=Microendoscopic Discectomy.MIS-TLIF=Minimally Invasive transforaminal lumbar interbody fusion. OD= Open discectomy.PELD=Percutaneous Endoscopic Lumbar Diskectomy. PLIF= posterior lumbar interbody fusion.TLIF= transforaminal lumbar interbody fusion.Unilat TLIF= Unilateral transforaminal lumbar interbody fusion.

| Comparison | Direct | Indirect | Network | P.value |
| --- | --- | --- | --- | --- |
| PELD.MED | -0.72 (-5.0, 3.6) | 1.7 (-50., 66.) | -0.72 (-5.1, 3.6) | 0.93 |
| MED.MIS-TLIF | -30. (-1.1e+02, -3.9) | -32. (-1.2e+02, 61.) | -41. (-1.0e+02, -7.1) | 0.97 |
| MED.PLIF | -30. ( -93., -3.5) | -34. (-1.2e+02, 0.13) | -23. (-79., -3.4) | 0.97 |
| PELD.MIS-TLIF | -33. (-1.1e+02, -5.8) | -43. (-1.5e+02, 58.) | -42. (-1.0e+02, -8.1) | 0.98 |
| PELD.OD | -0.47 (-5., 3.0) | -6.2 ( -82., 53.) | -0.50 (-5., 3.0) | 0.86 |
| PLIF.OD | 20. (0.14, 74.) | 32. (2.9, 1.1e+02) | 23. (3.2, 79.) | 0.69 |
