# Supplementary material for: Comparison of 7 surgical interventions for recurrent lumbar disc herniation: A network meta-analysis and systematic review
Source: PLoS One. 2025 Mar 4;20(3):e0309343. doi: 10.1371/journal.pone.0309343 (PMC11878942; doi:10.1371/journal.pone.0309343)
Supplement: S2 Table — (DOCX) [file pone.0309343.s003.docx]

Table 1. Rank possibility of VAS(back pain).

MED=Microendoscopic Discectomy.MIS-TLIF=Minimally Invasive transforaminal lumbar interbody fusion. OD= Open discectomy.PELD=Percutaneous Endoscopic Lumbar Diskectomy. PLIF= posterior lumbar interbody fusion.TLIF= transforaminal lumbar interbody fusion.Unilat TLIF= Unilateral transforaminal lumbar interbody fusion. VAS =visual analogue scale.

| Intervention | Rank 1 | Rank 2 | Rank 3 | | Rank 4 | | Rank 5 | Rank 6 | Rank 7 |
| --- | --- | --- | --- | --- | --- | --- | --- | --- | --- |
| MED | 0.02 | 0.04 | 0.08 | 0.09 | | 0.10 | | 0.13 | 0.53 |
| MIS_TLIF | 0.17 | 0.44 | 0.22 | 0.10 | | 0.05 | | 0.02 | 0.00 |
| OD | 0.01 | 0.03 | 0.09 | 0.19 | | 0.29 | | 0.27 | 0.13 |
| PELD | 0.01 | 0.04 | 0.13 | 0.21 | | 0.25 | | 0.27 | 0.10 |
| PLIF | 0.02 | 0.10 | 0.15 | 0.19 | | 0.16 | | 0.19 | 0.18 |
| TLIF | 0.01 | 0.23 | 0.29 | 0.18 | | 0.13 | | 0.10 | 0.05 |
| Unilat_TLIF | 0.77 | 0.12 | 0.05 | 0.02 | | 0.02 | | 0.01 | 0.01 |

Table 2. Rank possibility of VAS(leg pain)

| Intervention | Rank 1 | Rank 2 | Rank 3 |
| --- | --- | --- | --- |
| MED | 0.06 | 0.09 | 0.86 |
| MIS_TLIF | 0.42 | 0.52 | 0.06 |
| PELD | 0.53 | 0.40 | 0.08 |

MED=Microendoscopic Discectomy.MIS-TLIF=Minimally Invasive transforaminal lumbar interbody fusion. PELD=Percutaneous Endoscopic Lumbar Diskectomy. VAS =visual analogue scale.

Table 3. Rank possibility of ODI

MED=Microendoscopic Discectomy.MIS-TLIF=Minimally Invasive transforaminal lumbar interbody fusion. OD= Open discectomy.PELD=Percutaneous Endoscopic Lumbar Diskectomy. PLIF= posterior lumbar interbody fusion.TLIF= transforaminal lumbar interbody fusion.Unilat TLIF= Unilateral transforaminal lumbar interbody fusion.

ODI= Oswestry disability index.

| Intervention | Rank 1 | Rank 2 | Rank 3 | Rank 4 | Rank 5 | Rank 6 |
| --- | --- | --- | --- | --- | --- | --- |
| MED | 0.36 | 0.19 | 0.18 | 0.13 | 0.05 | 0.09 |
| MIS_TLIF | 0.00 | 0.01 | 0.05 | 0.18 | 0.35 | 0.40 |
| OD | 0.16 | 0.45 | 0.33 | 0.05 | 0.01 | 0.00 |
| PELD | 0.00 | 0.01 | 0.07 | 0.27 | 0.45 | 0.19 |
| PLIF | 0.01 | 0.07 | 0.21 | 0.31 | 0.11 | 0.29 |
| TLIF | 0.47 | 0.26 | 0.16 | 0.05 | 0.04 | 0.02 |

Table 4. Rank possibility of complication

MED=Microendoscopic Discectomy.MIS-TLIF=Minimally Invasive transforaminal lumbar interbody fusion. OD= Open discectomy.PELD=Percutaneous Endoscopic Lumbar Diskectomy. PLIF= posterior lumbar interbody fusion.TLIF= transforaminal lumbar interbody fusion.Unilat TLIF= Unilateral transforaminal lumbar interbody fusion.

| Intervention | Rank 1 | Rank 2 | Rank 3 | Rank 4 | Rank 5 | Rank 6 | Rank 7 |
| --- | --- | --- | --- | --- | --- | --- | --- |
| MED | 0.58 | 0.28 | 0.08 | 0.04 | 0.01 | 0.00 | 0.00 |
| MIS_TLIF | 0.00 | 0.00 | 0.01 | 0.04 | 0.37 | 0.45 | 0.13 |
| OD | 0.09 | 0.30 | 0.34 | 0.20 | 0.06 | 0.01 | 0.00 |
| PELD | 0.04 | 0.18 | 0.26 | 0.37 | 0.14 | 0.02 | 0.00 |
| PLIF | 0.03 | 0.22 | 0.30 | 0.31 | 0.12 | 0.02 | 0.00 |
| TLIF | 0.00 | 0.00 | 0.01 | 0.04 | 0.28 | 0.50 | 0.17 |
| Unilat_TLIF | 0.26 | 0.01 | 0.01 | 0.01 | 0.01 | 0.01 | 0.69 |

Table 5. Rank possibility of recurrence rate

MED=Microendoscopic Discectomy.MIS-TLIF=Minimally Invasive transforaminal lumbar interbody fusion. OD= Open discectomy.PELD=Percutaneous Endoscopic Lumbar Diskectomy. PLIF= posterior lumbar interbody fusion.TLIF= transforaminal lumbar interbody fusion.Unilat TLIF= Unilateral transforaminal lumbar interbody fusion.

| Intervention | Rank 1 | Rank 2 | Rank 3 | Rank 4 | Rank 5 | Rank 6 |
| --- | --- | --- | --- | --- | --- | --- |
| MED | 0.25 | 0.26 | 0.47 | 0.02 | 0.00 | 0.00 |
| MIS_TLIF | 0.00 | 0.00 | 0.00 | 0.24 | 0.23 | 0.52 |
| OD | 0.32 | 0.28 | 0.39 | 0.01 | 0.00 | 0.00 |
| PELD | 0.42 | 0.45 | 0.12 | 0.00 | 0.00 | 0.00 |
| PLIF | 0.00 | 0.00 | 0.00 | 0.35 | 0.43 | 0.22 |
| TLIF | 0.01 | 0.01 | 0.01 | 0.38 | 0.34 | 0.26 |
