# Supplementary material for: Comparison of 7 surgical interventions for recurrent lumbar disc herniation: A network meta-analysis and systematic review
Source: PLoS One. 2025 Mar 4;20(3):e0309343. doi: 10.1371/journal.pone.0309343 (PMC11878942; doi:10.1371/journal.pone.0309343)
Supplement: S3 Table — (DOCX) [file pone.0309343.s004.docx]

Table 1. Heterogeneity analysis of VAS(back pain)

MED=Microendoscopic Discectomy.MIS-TLIF=Minimally Invasive transforaminal lumbar interbody fusion. OD= Open discectomy.PELD=Percutaneous Endoscopic Lumbar Diskectomy. PLIF= posterior lumbar interbody fusion.TLIF= transforaminal lumbar interbody fusion.Unilat TLIF= Unilateral transforaminal lumbar interbody fusion. VAS =visual analogue scale.NA= Not Applicable.

| t1 | t2 | I^2^.pair(%) | I^2^.cons(%) | Incons.p |
| --- | --- | --- | --- | --- |
| MED | MIS_TLIF | 93.25 | 90.10 | NA |
| MED | PELD | NA | 0.00 | 0.76 |
| MIS_TLIF | PELD | 98.52 | 98.04 | 0.98 |
| MIS_TLIF | TLIF | NA | 0.00 | 0.75 |
| OD | PELD | 0.00 | 0.00 | 0.79 |
| OD | PLIF | 0.00 | 0.00 | NA |
| OD | TLIF | 0.00 | 0.00 | 0.79 |
| PLIF | TLIF | 0.00 | 0.00 | 0.84 |
| TLIF | Unilat_TLIF | 97.67 | 97.67 | NA |

Table 2. Heterogeneity analysis of VAS(leg pain)

| t1 | t2 | I^2^.pair(%) | I^2^.cons(%) | Incons.p |
| --- | --- | --- | --- | --- |
| MED | MIS_TLIF | 96.99 | 97.39 | NA |
| MED | PELD | NA | 61.77 |  |
| MIS_TLIF | PELD | 55.63 | 45.00 | NA |

MED=Microendoscopic Discectomy.MIS-TLIF=Minimally Invasive transforaminal lumbar interbody fusion. PELD=Percutaneous Endoscopic Lumbar Diskectomy. VAS =visual analogue scale. NA= Not Applicable.

Table 3. Heterogeneity analysis of ODI

| t1 | t2 | I^2^.pair(%) | I^2^.cons(%) | Incons.p |
| --- | --- | --- | --- | --- |
| MED | MIS_TLIF | NA | NA | NA |
| MED | PELD | NA | NA | NA |
| MIS_TLIF | PELD | 0.00 | 0.00 | NA |
| OD | PELD | 0.00 | 0.00 | NA |
| OD | PLIF | 6.90 | 9.90 | NA |
| OD | TLIF | 0.00 | 0.00 | NA |
| PLIF | TLIF | 0.00 | 0.00 | NA |

MED=Microendoscopic Discectomy.MIS-TLIF=Minimally Invasive transforaminal lumbar interbody fusion. OD= Open discectomy.PELD=Percutaneous Endoscopic Lumbar Diskectomy. PLIF= posterior lumbar interbody fusion.TLIF= transforaminal lumbar interbody fusion.Unilat TLIF= Unilateral transforaminal lumbar interbody fusion.ODI= Oswestry disability index. NA= Not Applicable.

Table 4. Heterogeneity analysis of recurrence rate

MED=Microendoscopic Discectomy.MIS-TLIF=Minimally Invasive transforaminal lumbar interbody fusion. OD= Open discectomy.PELD=Percutaneous Endoscopic Lumbar Diskectomy. PLIF= posterior lumbar interbody fusion.TLIF= transforaminal lumbar interbody fusion.Unilat TLIF= Unilateral transforaminal lumbar interbody fusion. NA= Not Applicable.

| t1 | t2 | I^2^.pair(%) | I^2^.cons(%) | Incons.p |
| --- | --- | --- | --- | --- |
| MED | MIS_TLIF | 0.00 | 0.00 | 0.46 |
| MED | PELD | NA | 0.00 | 1.00 |
| MED | PLIF | NA | 0.00 | 0.20 |
| MIS_TLIF | PELD | 0.00 | 0.00 | 0.16 |
| OD | PELD | 96.75 | 24.32 | 0.80 |
| OD | PLIF | NA | 0.00 | 0.61 |
| OD | TLIF | NA | NA | NA |
| PLIF | TLIF | NA | NA | NA |

Table 5. Heterogeneity analysis of complication

MED=Microendoscopic Discectomy.MIS-TLIF=Minimally Invasive transforaminal lumbar interbody fusion. OD= Open discectomy.PELD=Percutaneous Endoscopic Lumbar Diskectomy. PLIF= posterior lumbar interbody fusion.TLIF= transforaminal lumbar interbody fusion.Unilat TLIF= Unilateral transforaminal lumbar interbody fusion. NA= Not Applicable.

| t1 | t2 | I^2^.pair(%) | I^2^.cons(%) | Incons.p |
| --- | --- | --- | --- | --- |
| MED | MIS_TLIF | 10.18 | 0.00 | 0.47 |
| MED | PELD | NA | 0.00 | 1.00 |
| MED | PLIF | NA | 0.00 | 0.32 |
| MIS_TLIF | PELD | 0.00 | 0.00 | 0.26 |
| OD | PELD | 96.61 | 23.01 | 0.80 |
| OD | PLIF | NA | 0.00 | 0.63 |
| OD | TLIF | NA | NA | NA |
| PLIF | TLIF | NA | NA | NA |
