# Supplementary material for: Comparison of 7 surgical interventions for recurrent lumbar disc herniation: A network meta-analysis and systematic review
Source: PLoS One. 2025 Mar 4;20(3):e0309343. doi: 10.1371/journal.pone.0309343 (PMC11878942; doi:10.1371/journal.pone.0309343)
Supplement: S4 Table — (DOCX) [file pone.0309343.s005.docx]

**Table 1.** Meta-analysis data of the study (VAS(back pain))

| **study** | **treatment** | **mean** | **std.dev** | **sampleSize** |
| --- | --- | --- | --- | --- |
| Anqi Wang et al. (2020) | PELD | -5.85 | 0.76 | 24 |
| Anqi Wang et al. (2020) | MIS-TLIF | -6.30 | 0.77 | 22 |
| Yuan Yao et al. (2017) | PELD | -2.86 | 1.45 | 28 |
| Yuan Yao et al. (2017) | MIS-TLIF | -2.04 | 1.40 | 26 |
| Yuan Yao et al. (2017) | MED | -2.26 | 1.68 | 20 |
| Salvatore D’Oria et al. (2023) | MIS-TLIF | -6.10 | 0.85 | 45 |
| Salvatore D’Oria et al. (2023) | MED | -4.62 | 1.02 | 45 |
| Junlong Wu et al. (2017) | PELD | -3.44 | 1.32 | 47 |
| Junlong Wu et al. (2017) | MIS-TLIF | -3.72 | 1.21 | 58 |
| Chao Liu et al. (2024) | PELD | -0.70 | 1.26 | 209 |
| Chao Liu et al. (2024) | MIS-TLIF | -3.00 | 1.15 | 192 |
| Erkin Sonmez et al. (2013) | Unilat TLIF | -6.70 | 0.73 | 10 |
| Erkin Sonmez et al. (2013) | TLIF | -6.70 | 0.57 | 10 |
| Xianglong Zhuo et al. (2009） | OD | -5.10 | 1.46 | 25 |
| Xianglong Zhuo et al. (2009) | TLIF | -5.40 | 1.37 | 18 |
| Xianglong Zhuo et al. (2009) | PLIF | -4.90 | 1.47 | 22 |
| Yongsheng Hu et al. (2023) | OD | -5.25 | 1.10 | 31 |
| Yongsheng Hu et al. (2023) | PLIF | -5.33 | 1.10 | 42 |
| Junhai Lu et al. (2022) | PELD | -7.57 | 1.64 | 56 |
| Junhai Lu et al. (2022) | OD | -7.58 | 1.88 | 58 |
| Hao Xue (2016) | PELD | -5.60 | 2.11 | 18 |
| Hao Xue (2016) | OD | -5.84 | 2.07 | 18 |
| Xiaogang Hu  (2017) | PELD | -8.22 | 1.49 | 53 |
| Xiaogang Hu  (2017) | OD | -8.10 | 1.52 | 37 |
| Tianji Zhang et al. (2017) | PELD | -8.40 | 0.37 | 41 |
| Tianji Zhang et al. (2017) | OD | -8.20 | 0.46 | 41 |
| Jiancheng Su et al. (2016) | PELD | -8.41 | 0.62 | 36 |
| Jiancheng Su et al. (2016) | OD | -8.18 | 0.67 | 40 |
| Yinhe Chen et al. (2014) | OD | -5.30 | 0.86 | 12 |
| Yinhe Chen et al. (2014) | TLIF | -5.50 | 0.80 | 26 |
| Yinhe Chen et al. (2014) | PLIF | -5.50 | 0.83 | 27 |
| Guiying Gao et al. (2019) | MIS-TLIF | -5.75 | 1.20 | 34 |
| Guiying Gao et al. (2019) | TLIF | -5.67 | 1.17 | 34 |
| Bing Pan et al. (2014) | Unilat TLIF | -4.03 | 0.73 | 26 |
| Bing Pan et al. (2014) | TLIF | -2.06 | 0.46 | 25 |

**Table 2.** Meta-analysis data of the study (VAS(leg pain))

| **study** | **treatment** | **mean** | **std.dev** | **sampleSize** |
| --- | --- | --- | --- | --- |
| Anqi Wang et al. (2020) | PELD | -6.05 | 0.72 | 24 |
| Anqi Wang et al. (2020) | MIS-TLIF | -6.1 | 0.7 | 22 |
| Yuan Yao et al. (2017) | PELD | -2.44 | 1.1 | 28 |
| Yuan Yao et al. (2017) | MIS-TLIF | -1.58 | 1.55 | 26 |
| Yuan Yao et al. (2017) | MED | -1.96 | 1.27 | 20 |
| Salvatore D’Oria et al. (2023) | MIS-TLIF | -4.5 | 0.93 | 45 |
| Salvatore D’Oria et al. (2023) | MED | -2.33 | 0.97 | 45 |
| Junlong Wu et al. (2017) | PELD | -5.3 | 1.06 | 47 |
| Junlong Wu et al. (2017) | MIS-TLIF | -5.43 | 1.03 | 58 |
| Chao Liu et al. (2024) | PELD | -5 | 2.02 | 209 |
| Chao Liu et al. (2024) | MIS-TLIF | -4.7 | 2.12 | 92 |
| Erkin Sonmez et al. (2013) | Unilat TLIF | -6.05 | 0.5 | 10 |
| Erkin Sonmez et al. (2013) | TLIF | -6.1 | 0.68 | 10 |
| Bing Pan et al. (2014) | Unilat TLIF | -5.76 | 0.36 | 26 |
| Bing Pan et al. (2014) | TLIF | -5.9 | 0.99 | 25 |

**Table 3.** Meta-analysis data of the study (ODI)

| **study** | **trt** | **mean** | **sd** | **n** |
| --- | --- | --- | --- | --- |
| Anqi Wang et al. (2020) | PELD | -17.5 | 1.55 | 24 |
| Anqi Wang et al. (2020) | MIS-TLIF | -17.5 | 1.84 | 22 |
| Yuan Yao et al. (2017) | PELD | -11.39 | 5.46 | 28 |
| Yuan Yao et al. (2017) | MIS-TLIF | -12 | 4.61 | 26 |
| Yuan Yao et al. (2017) | MED | -13.77 | 6.84 | 20 |
| Yuan Yao et al. (2017) | PELD | -16.04 | 4.36 | 47 |
| Yuan Yao et al. (2017) | MIS-TLIF | -15.93 | 3.32 | 58 |
| Chao Liu et al. (2024) | PELD | -27.3 | 4.90 | 209 |
| Chao Liu et al. (2024) | MIS-TLIF | -26.8 | 5.39 | 192 |
| Xianglong Zhuo et al. (2009) | OD | -42.5 | 5.07 | 25 |
| Xianglong Zhuo et al. (2009) | TLIF | -43.4 | 5.15 | 18 |
| Xianglong Zhuo et al. (2009) | PLIF | -41.5 | 4.52 | 22 |
| Yongsheng Hu et al. (2023) | OD | -34.08 | 7.18 | 31 |
| Yongsheng Hu et al. (2023) | PLIF | -30.74 | 7.61 | 42 |
| Junhai Lu et al. (2022) | PELD | -64.8 | 21.00 | 56 |
| Junhai Lu et al. (2022) | OD | -63.69 | 21.25 | 58 |
| Xiaogang Hu  (2017) | PELD | -37.26 | 7.17 | 53 |
| Xiaogang Hu  (2017) | OD | -38.89 | 6.50 | 37 |
| Tianji Zhang et al. (2017) | PELD | -37.4 | 5.32 | 41 |
| Tianji Zhang et al. (2017) | OD | -39.8 | 3.96 | 41 |
| Jiancheng Su et al. (2016) | PELD | -38.03 | 7.35 | 36 |
| Jiancheng Su et al. (2016) | OD | -39.66 | 5.75 | 40 |
| Yinhe Chen et al. (2014) | OD | -46.5 | 10.17 | 12 |
| Yinhe Chen et al. (2014) | TLIF | -49.9 | 9.13 | 26 |
| Yinhe Chen et al. (2014) | PLIF | -50 | 11.09 | 27 |

**Table 4.** Meta-analysis data of the study (Complication)

| **study** | **trt** | **d** | **n** |
| --- | --- | --- | --- |
| Anqi Wang et al. (2020) | PELD | 2 | 24 |
| Anqi Wang et al. (2020) | MIS-TLIF | 1 | 22 |
| Yuan Yao et al. (2017) | PELD | 4 | 28 |
| Yuan Yao et al. (2017) | MIS-TLIF | 1 | 26 |
| Yuan Yao et al. (2017) | MED | 2 | 20 |
| Gerald Musa et al. (2024) | PLIF | 4 | 34 |
| Gerald Musa et al. (2024) | MED | 15 | 40 |
| Junlong Wu et al. (2017) | PELD | 4 | 47 |
| Junlong Wu et al. (2017) | MIS-TLIF | 1 | 58 |
| Chao Liu et al. (2024) | PELD | 23 | 209 |
| Chao Liu et al. (2024) | MIS-TLIF | 11 | 192 |
| Ahmed Zaater et al. (2016) | OD | 5 | 24 |
| Ahmed Zaater et al. (2016) | PLIF | 5 | 15 |
| Erkin Sonmez et al. (2013) | Unilat TLIF | 0 | 10 |
| Erkin Sonmez et al. (2013) | TLIF | 0 | 10 |
| Xianglong Zhuo et al. (2009) | OD | 6 | 25 |
| Xianglong Zhuo et al. (2009) | TLIF | 1 | 18 |
| Xianglong Zhuo et al. (2009) | PLIF | 5 | 22 |
| Junhai Lu et al. (2022) | PELD | 2 | 56 |
| Junhai Lu et al. (2022) | OD | 3 | 58 |
| Hao Xue (2016) | PELD | 2 | 18 |
| Hao Xue (2016) | OD | 2 | 18 |
| Xiaogang Hu  (2017) | PELD | 1 | 53 |
| Xiaogang Hu  (2017) | OD | 2 | 37 |
| Jiancheng Su et al. (2016) | PELD | 1 | 36 |
| Jiancheng Su et al. (2016) | OD | 2 | 40 |
| Yinhe Chen et al. (2014) | OD | 1 | 12 |
| Yinhe Chen et al. (2014) | TLIF | 1 | 26 |
| Yinhe Chen et al. (2014) | PLIF | 1 | 27 |
| Guiying Gao et al. (2019) | MIS-TLIF | 4 | 34 |
| Guiying Gao et al. (2019) | TLIF | 5 | 34 |
| Liqiang Li et al. (2016) | TLIF | 2 | 26 |
| Liqiang Li et al. (2016) | PLIF | 7 | 25 |

**Table 5.** Meta-analysis data of the study (Recurrence)

| **study** | **trt** | **d** | **n** |
| --- | --- | --- | --- |
| Anqi Wang et al. (2020) | PELD | 5 | 24 |
| Anqi Wang et al. (2020) | MIS-TLIF | 0 | 22 |
| Yuan Yao et al. (2017) | PELD | 7 | 28 |
| Yuan Yao et al. (2017) | MIS-TLIF | 0 | 26 |
| Yuan Yao et al. (2017) | MED | 3 | 20 |
| Salvatore D’Oria et al. (2023) | MIS-TLIF | 0 | 45 |
| Salvatore D’Oria et al. (2023) | MED | 6 | 45 |
| Gerald Musa et al. (2024) | PLIF | 0 | 34 |
| Gerald Musa et al. (2024) | MED | 9 | 40 |
| Junlong Wu et al. (2017) | PELD | 5 | 47 |
| Junlong Wu et al. (2017) | MIS-TLIF | 0 | 58 |
| Chao Liu et al. (2024) | PELD | 12 | 209 |
| Chao Liu et al. (2024) | MIS-TLIF | 0 | 192 |
| Ayman A et al. (2013) | OD | 1 | 15 |
| Ayman A et al. (2013) | TLIF | 0 | 15 |
| Ayman A et al. (2013) | PLIF | 0 | 15 |
| Junhai Lu et al. (2022) | PELD | 1 | 56 |
| Junhai Lu et al. (2022) | OD | 0 | 58 |
| Jiancheng Su et al. (2016) | PELD | 1 | 36 |
| Jiancheng Su et al. (2016) | OD | 2 | 40 |
