# Supplementary material for: Comparison of 7 surgical interventions for recurrent lumbar disc herniation: A network meta-analysis and systematic review
Source: PLoS One. 2025 Mar 4;20(3):e0309343. doi: 10.1371/journal.pone.0309343 (PMC11878942; doi:10.1371/journal.pone.0309343)
Supplement: S5 Table — (DOCX) [file pone.0309343.s006.docx]

**Table 1** Raw data for this study(VAS ,visual analogue scale)

|  |  |  |  | **Treatments** | | |  | **VAS** | | | | | | | | | | | |
| --- | --- | --- | --- | --- | --- | --- | --- | --- | --- | --- | --- | --- | --- | --- | --- | --- | --- | --- | --- |
|  |  |  |  |  |  |  |  | Group 1 | | | | Group 2 | | | | Group 3 | | | |
|  | Authors and years | data extractor | Date of data extraction | Group 1 | Group 2 | Group 3 | Inclusion in analysis | back pain | | leg pain | | back pain | | leg pain | | back pain | | leg pain | |
|  |  |  |  |  |  |  |  | Preoperative | postoperation 12 months | Preoperative | postoperation 12 months | Preoperative | postoperation 12 months | Preoperative | postoperation 12 months | Preoperative | postoperation 12 months | Preoperative | postoperation 12 months |
| 1 | Anqi Wang et al. (2020) | Hang Zhang | April 22th, 2024 | PELD | MIS-TLIF |  | Yes | 7.05±0.76 | 1.20±0.62 | 7.15±0.67 | 1.10±0.64 | 7.20±0.79 | 0.90±0.57 | 7.10±0.74 | 1.00±0.47 |  |  |  |  |
| 2 | Yuan Yao et al. (2017) | Hang Zhang | April 22th, 2024 | PELD | MIS-TLIF | MED | Yes | 5.86±1.11 | 3.00 ±1.48 | 7.22 ±1.00 | 4.78±1.00 | 5.96 ±1.15 | 3.92±1.38 | 6.96±1.28 | 5.38 ±1.53 | 6.20 ±1.24 | 3.94±1.73 | 7.35±0.99 | 5.39 ±1.29 |
| 3 | Salvatore D’Oria et al. (2023) | Hang Zhang | April 22th, 2024 |  | MIS-TLIF | MED | Yes |  |  |  |  | 7.53 ±0.89 | 1.43±0.12 | 6.76±0.99 | 2, 26 ±0.19 | 7.72±1.11 | 3.10 ±0.45 | 6.57±1.04 | 4.24±0.25 |
| 4 | Gerald Musa et al. (2024) | Hang Zhang | April 22th, 2024 |  | PLIF | MED | Yes |  |  |  |  |  |  |  |  |  |  |  |  |
| 5 | Junlong Wu et al. (2017) | Hang Zhang | April 22th, 2024 | PELD | MIS-TLIF |  | Yes | 5.91±1.33 | 2.468±1.04 | 7.13±1.10 | 1.83±0.76 | 5.88±1.24 | 2.16±0.90 | 7.05±1.08 | 1.62±0.70 |  |  |  |  |
| 6 | Chao Liu et al. (2024) | Hang Zhang | April 22th, 2024 | PELD | MIS-TLIF |  | Yes | 3.8±1.1 | 3.1±1.2 | 6.1 ±2.2 | 1.1±0.8 | 4.4±1.2 | 1.4 ±0.8 | 5.9± 2.3 | 1.2±0.7 |  |  |  |  |
| 7 | Ayman A et al. (2013) | Hang Zhang | April 22th, 2024 | OD | TLIF | PLIF | Yes |  |  |  |  |  |  |  |  |  |  |  |  |
| 8 | Ahmed Zaater et al. (2016) | Hang Zhang | April 22th, 2024 | OD | PLIF |  | Yes |  |  |  |  |  |  |  |  |  |  |  |  |
| 9 | Erkin Sonmez et al. (2013) | Hang Zhang | April 22th, 2024 |  | Unilat TLIF | TLIF | Yes |  |  |  |  | 8.5±0.8 | 1.8±0.3 | 8.45±0.5 | 2.4±0.4 | 8.4±0.6 | 1.7±0.4 | 8.6±0.7 | 2.5±0.5 |
| 10 | Xianglong Zhuo et al. (2009) | Hang Zhang | April 22th, 2024 | OD | TLIF | PLIF | Yes | 7.2±1.53 | 2.1±1.02 |  |  | 7.4±1.46 | 2.0±0.90 |  |  | 7.1±1.55 | 2.2±1.01 |  |  |
| 11 | Yongsheng Hu et al. (2023) | QiPeng Xie | April 22th, 2024 | OD |  | PLIF | Yes | 7.46±1.01 | 2.21±1.00 |  |  |  |  |  |  | 7.68±1.03 | 2.35±0.97 |  |  |
| 12 | Junhai Lu et al. (2022) | QiPeng Xie | April 22th, 2024 | PELD |  | OD | Yes | 7.57 ± 1.64 | 0.00 ± 0.00 |  |  |  |  |  |  | 7.68 ± 1.98 | 0.10 ± 0.30 |  |  |
| 13 | Hao Xue (2016) | QiPeng Xie | April 22th, 2024 | PELD |  | OD | Yes | 6.02±2.16 | 0.42±0.13 |  |  |  |  |  |  | 6.14±2.10 | 0.30±0.08 |  |  |
| 14 | Xiaogang Hu  (2017) | QiPeng Xie | April 22th, 2024 | PELD |  | OD | Yes | 8.65±1.57 | 0.43±0.26 |  |  |  |  |  |  | 8.57±1.62 | 0.47±0.30 |  |  |
| 15 | Tianji Zhang et al. (2017) | QiPeng Xie | April 22th, 2024 | PELD |  | OD | Yes | 8. 7 ± 0. 4 | 0. 3 ± 0. 2 |  |  |  |  |  |  | 8. 6 ± 0. 5 | 0. 4 ± 0. 2 |  |  |
| 16 | Jiancheng Su et al. （2016） | QiPeng Xie | April 22th, 2024 | PELD |  | OD | Yes | 8.73 ± 0.64 | 0.32 ± 0.47 |  |  |  |  |  |  | 8.59 ± 0.72 | 0.41 ± 0.39 |  |  |
| 17 | Yinhe Chen et al. (2014) | QiPeng Xie | April 22th, 2024 | OD | TLIF | PLIF | Yes | 6.6 ± 0.79 | 1.3 ± 0.78 |  |  | 6.4 ± 0.68 | 0.9 ± 0.77 |  |  | 6.5 ± 0.80 | 1.0 ± 0.71 |  |  |
| 18 | Guiying Gao et al. (2019) | QiPeng Xie | April 22th, 2024 |  | MIS-TLIF | TLIF | Yes |  |  |  |  | 7.34 ± 1.28 | 1.59 ± 0.26 |  |  | 7.29 ± 1.25 | 1.62 ± 0.24 |  |  |
| 19 | Liqiang Li et al. (2016) | QiPeng Xie | April 22th, 2024 |  | TLIF | PLIF | Yes |  |  |  |  |  |  |  |  |  |  |  |  |
| 20 | Bing Pan et al. (2014) | QiPeng Xie | April 22th, 2024 |  | Unilat TLIF | TLIF | Yes |  |  |  |  | 4.93 ± 0.65 | 0.90±0.69 | 7.13±0.36 | 1.37±0.29 | 5.13±0.38 | 3.07±0.46 | 7.35±1.08 | 1.45±0.36 |

**Table 2.** Raw data for this study(**ODI ,Oswestry disability index)**

|  |  |  |  | **Treatments** | | |  | **ODI** | | | | | |
| --- | --- | --- | --- | --- | --- | --- | --- | --- | --- | --- | --- | --- | --- |
|  | Authors and years | data extractor | Date of data extraction | Group 1 | Group 2 | Group 3 | Inclusion in analysis | Group 1 | | Group 2 | | Group 3 | |
|  |  |  |  |  |  |  |  | Preoperative | postoperation 12 months | Preoperative | postoperation 12 months | Preoperative | postoperation 12 months |
| 1 | Anqi Wang et al. (2020) | Hang Zhang | April 22th, 2024 | PELD | MIS-TLIF |  | Yes | 28.15±1.69 | 10.65±0.81 | 28.30±2.00 | 10.80±0.63 |  |  |
| 2 | Yuan Yao et al. (2017) | Hang Zhang | April 22th, 2024 | PELD | MIS-TLIF | MED | Yes | 27.65±4.66 | 16.26±5.27 | 28.0±4.02 | 16.00±4.38 | 29.10±5.17 | 15.33±7.00 |
| 3 | Salvatore D’Oria et al. (2023) | Hang Zhang | April 22th, 2024 |  | MIS-TLIF | MED | Yes |  |  |  |  |  |  |
| 4 | Gerald Musa et al. (2024) | Hang Zhang | April 22th, 2024 |  | PLIF | MED | Yes |  |  |  |  |  |  |
| 5 | Junlong Wu et al. (2017) | Hang Zhang | April 22th, 2024 | PELD | MIS-TLIF |  | Yes | 28.55±4.73 | 12.51±2.34 | 27.76±3.61 | 11.83±1.69 |  |  |
| 6 | Chao Liu et al. (2024) | Hang Zhang | April 22th, 2024 | PELD | MIS-TLIF |  | Yes | 38.7±5.2 | 11.4±3.2 | 39.2±5.7 | 12.4 ±3.6 |  |  |
| 7 | Ayman A et al. (2013) | Hang Zhang | April 22th, 2024 | OD | TLIF | PLIF | Yes |  |  |  |  |  |  |
| 8 | Ahmed Zaater et al. (2016) | Hang Zhang | April 22th, 2024 | OD | PLIF |  | Yes |  |  |  |  |  |  |
| 9 | Erkin Sonmez et al. (2013) | Hang Zhang | April 22th, 2024 |  | Unilat TLIF | TLIF | Yes |  |  |  |  |  |  |
| 10 | Xianglong Zhuo et al. (2009) | Hang Zhang | April 22th, 2024 | OD | TLIF | PLIF | Yes | 54.4±5.5 | 11.9±2.7 | 55.1±5.6 | 11.7±1.8 | 53.5±4.9 | 12.0±2.5 |
| 11 | Yongsheng Hu et al. (2023) | QiPeng Xie | April 22th, 2024 | OD |  | PLIF | Yes | 48.03±7.84 | 13.95±3.38 |  |  | 47.32±7.95 | 16.58±5.37 |
| 12 | Junhai Lu et al. (2022) | QiPeng Xie | April 22th, 2024 | PELD |  | OD | Yes | 66.82 ± 21.86 | 2.02 ± 2.46 |  |  | 65.44 ± 22.14 | 1.75 ± 2.53 |
| 13 | Hao Xue (2016) | QiPeng Xie | April 22th, 2024 | PELD |  | OD | Yes |  |  |  |  |  |  |
| 14 | Xiaogang Hu  (2017) | QiPeng Xie | April 22th, 2024 | PELD |  | OD | Yes | 39.87±7.65 | 2.61±1.55 |  |  | 41.53±6.98 | 2.64±1.62 |
| 15 | Tianji Zhang et al. (2017) | QiPeng Xie | April 22th, 2024 | PELD |  | OD | Yes | 39. 8 ± 5. 7 | 2. 4 ± 1. 3 |  |  | 42. 3 ± 4. 3 | 2. 5 ± 1. 3 |
| 16 | Jiancheng Su et al. （2016） | QiPeng Xie | April 22th, 2024 | PELD |  | OD | Yes | 40.57 ± 7.89 | 2.54 ± 1.84 |  |  | 42.30 ± 6.23 | 2.64 ± 1.79 |
| 17 | Yinhe Chen et al. (2014) | QiPeng Xie | April 22th, 2024 | OD | TLIF | PLIF | Yes | 57.6 ± 10.5 | 11.1 ± 7.5 | 55.3 ± 9.9 | 5.4 ± 5.0 | 56.2 ± 12.1 | 6.2 ± 4.6 |
| 18 | Guiying Gao et al. (2019) | QiPeng Xie | April 22th, 2024 |  | MIS-TLIF | TLIF | Yes |  |  |  |  |  |  |
| 19 | Liqiang Li et al. (2016) | QiPeng Xie | April 22th, 2024 |  | TLIF | PLIF | Yes |  |  |  |  |  |  |
| 20 | Bing Pan et al. (2014) | QiPeng Xie | April 22th, 2024 |  | Unilat TLIF | TLIF | Yes |  |  |  |  |  |  |

**Table3.** Raw data for this study(**Complication and Recurrence)**

|  |  |  |  | **Treatments** | | |  |  |  |  |  |  |  |
| --- | --- | --- | --- | --- | --- | --- | --- | --- | --- | --- | --- | --- | --- |
|  | Authors and years | data extractor | Date of data extraction | Group 1 | Group 2 | Group 3 | Inclusion in analysis | **Complication, n (%)** | | | **Recurrence, n (%)** | | |
|  |  |  |  |  |  |  |  | Group 1 | Group 2 | Group 3 | Group 1 | Group 2 | Group 3 |
| 1 | Anqi Wang et al. (2020) | Hang Zhang | April 22th, 2024 | PELD | MIS-TLIF |  | Yes | 2 (8.33) | 1 (4.55) |  | 5 (20.83) | 0 (0) |  |
| 2 | Yuan Yao et al. (2017) | Hang Zhang | April 22th, 2024 | PELD | MIS-TLIF | MED | Yes | 4 (14.29) | 1 (3.85) | 2 (10.00) | 7 (25.00) | 0 (0) | 3 (15.00) |
| 3 | Salvatore D’Oria et al. (2023) | Hang Zhang | April 22th, 2024 |  | MIS-TLIF | MED | Yes |  |  |  |  | 0 (0) | 6（13.33） |
| 4 | Gerald Musa et al. (2024) | Hang Zhang | April 22th, 2024 |  | PLIF | MED | Yes |  | 4(11.8) | 15(37.5) |  | 0 (0) | 9(22.5) |
| 5 | Junlong Wu et al. (2017) | Hang Zhang | April 22th, 2024 | PELD | MIS-TLIF |  | Yes | 4 (8.51) | 1 (1.72) |  | 5 (10.64) | 0 (0) |  |
| 6 | Chao Liu et al. (2024) | Hang Zhang | April 22th, 2024 | PELD | MIS-TLIF |  | Yes | 23(11.00) | 11(6.25) |  | 12(5.74) | 0 (0) |  |
| 7 | Ayman A et al. (2013) | Hang Zhang | April 22th, 2024 | OD | TLIF | PLIF | Yes |  |  |  | 1 (6.7) | 0 (0) | 0 (0) |
| 8 | Ahmed Zaater et al. (2016) | Hang Zhang | April 22th, 2024 | OD | PLIF |  | Yes | 5 (20) | 5（30） |  |  |  |  |
| 9 | Erkin Sonmez et al. (2013) | Hang Zhang | April 22th, 2024 |  | Unilat-TLIF | TLIF | Yes |  | 0 (0.0) | 0 (0.0) |  |  |  |
| 10 | Xianglong Zhuo et al. (2009) | Hang Zhang | April 22th, 2024 | OD | TLIF | PLIF | Yes | 6（24） | 1（5.6） | 5（22.3） |  |  |  |
| 11 | Yongsheng Hu et al. (2023) | QiPeng Xie | April 22th, 2024 | OD |  | PLIF | Yes |  |  |  |  |  |  |
| 12 | Junhai Lu et al. (2022) | QiPeng Xie | April 22th, 2024 | PELD |  | OD | Yes | 2(3.57) |  | 3(5.17) | 1( 1.785) |  | 0(0) |
| 13 | Hao Xue (2016) | QiPeng Xie | April 22th, 2024 | PELD |  | OD | Yes | 2（11.11） |  | 2（11.11） |  |  |  |
| 14 | Xiaogang Hu  (2017) | QiPeng Xie | April 22th, 2024 | PELD |  | OD | Yes | 1(1.8) |  | 2(5.4) |  |  |  |
| 15 | Tianji Zhang et al. (2017) | QiPeng Xie | April 22th, 2024 | PELD |  | OD | Yes |  |  |  |  |  |  |
| 16 | Jiancheng Su et al. (2016) | QiPeng Xie | April 22th, 2024 | PELD |  | OD | Yes |  | 1(2.78) |  | 1(2.78) |  | 2(5.00) |
| 17 | Yinhe Chen et al. (2014) | QiPeng Xie | April 22th, 2024 | OD | TLIF | PLIF | Yes | 1(8.33) | 1(3.85) | 1(3.70) |  |  |  |
| 18 | Guiying Gao et al. (2019) | QiPeng Xie | April 22th, 2024 |  | MIS-TLIF | TLIF | Yes |  | 4(11. 76) | 5(14. 71) |  |  |  |
| 19 | Liqiang Li et al. (2016) | QiPeng Xie | April 22th, 2024 |  | TLIF | PLIF | Yes |  | 2(8.00) | 7(26.92) |  |  |  |
| 20 | Bing Pan et al. (2014) | QiPeng Xie | April 22th, 2024 |  | Unilat TLIF | TLIF | Yes |  |  |  |  |  |  |
