# Supplementary material for: Comparison of 7 surgical interventions for recurrent lumbar disc herniation: A network meta-analysis and systematic review
Source: PLoS One. 2025 Mar 4;20(3):e0309343. doi: 10.1371/journal.pone.0309343 (PMC11878942; doi:10.1371/journal.pone.0309343)
Supplement: S6 Table — (DOCX) [file pone.0309343.s007.docx]

Table 1. Sifting the list of qualified literature

| Number | Authors and years | Country | DOI | Exclusion of cause |
| --- | --- | --- | --- | --- |
| 1 | Hu YongSheng et al. (2023) | China | doi: 10.3969/j.issn.1674-3806.2023.11.14 | Wrong intervention |
| 2 | Yu Feng (2023) | China | doi:10.3969/j. issn.1672-0369.2023.05.046 | Duplicate |
| 3 | Lu Junhai et al. (2022) | China | doi: 10.3969/j.issn.1001-5639.2022.02.006 | Duplicate |
| 4 | He GuiHua (2022) | China | doi: 10.3969/j.issn.2095-7629.2022.13.021 | Wrong intervention |
| 5 | Xu Xiaoping et al. (2021) | China | doi:10.3969/j.issn.1005-7234.2021.01.016 | Duplicate |
| 6 | Ahsan et al. (2021) | Bangladesh | doi:10.4103/jcvjs.jcvjs_153_20 | Wrong intervention |
| 7 | Cui Wei et al. (2021) | China | doi：10.7531/j.issn.1672-9935.2021.09.004 | Wrong intervention |
| 8 | Shifeng Jiang et al. (2021) | China | doi:10.1097/MD.0000000000025806 | Duplicate |
| 9 | Ahmed et al. (2020) | Egypt | doi:10.1186/s41983-020-0161-0 | Wrong intervention |
| 10 | Wu BoYu et al. (2020) | China | doi:10.3977/j.issn.1005-8478.2020.01.03 | Duplicate |
| 11 | Du Feng et al. (2019) | China | doi:10.3969/j.issn.1005-7234.2019.03.038 | Duplicate |
| 12 | Han Jianguo et al. (2018) | China | doi: 10.11724/jdmu.2018.02.11 | Wrong intervention |
| 13 | Han Jianguo et al. (2018) | China | doi: 10.7683 /xxyxyxb.2018.06.008 | Wrong intervention |
| 14 | Jian Guan et al. (2017) | America | doi:10.3171/2016.5.SPINE1616 | Wrong outcomes |
| 15 | Xia YuDong et al. (2017) | China | doi: 10.3969/j.issn.1004-0188.2017.03.023 | Wrong outcomes |
| 16 | Qiu GongMing (2016) | China | doi:10.19381/j.issn.1001-7585.2016.19.034 | Duplicate |
| 17 | Luo Tianhang et al. (2016) | China | doi:10.7531/j.issn.1672-9935.2016.09.027 | Duplicate |
| 18 | Su JianChen et al. (2016) | China | doi:10.13795/j.cnki.sgkz.2016.03.018 | Duplicate |
| 19 | Zuo Yu et al.  (2015) | China | doi:10.3969/j.issn.1674-4659.2015.06.0737 | Duplicate |
| 20 | Zhao ChongRan et al. (2013) | China | doi: 10.3969/j.issn.1673-7210.2013.06.024 | Wrong outcomes |
| 21 | Zhang Wei et al. (2013) | China | doi:10.3977/j.issn.1005-8478.2013.09.07 | Lacking or with incomplete information |
| 22 | Yuan Zhenchao et al. (2013) | China | doi:10.3969/j.issn.2095-252X.2013.04.007 | Wrong intervention |
| 23 | Huang ChengJun et al. (2013) | China | doi:10.3969/j.issn.1003-0034.2013.10.004 | Wrong outcomes |
| 24 | Li Zhen  (2012) | China | doi:10.3969 /j.issn.1672-0369.2012.10.015 | Duplicate |
| 25 | Huang WeiMing et al. (2012) | China | doi:10.3977 /j.issn.1005-8478.2012.15.10 | Duplicate |
| 26 | Kasmu et al. (2012) | China | doi:10.15932/j.0253-9713.2012.01.019 | Duplicate |
| 27 | Duan YongJiang (2012) | China | doi: 10.3969/j.issn.1672-5042.2012.01.021 | Wrong intervention |
| 28 | Wang HuiXue et al. (2011) | China | doi:10.3877/cma.j.issn.1674-0785.2011.02.044 | Duplicate |
| 29 | Qiao Pan et al. (2011) | China | doi: 10.7666/d.y1900731 | Duplicate |
| 30 | Zhou YeYuan et al. (2010) | China | doi:10.13241/j.cnki.pmb.2010.18.036 | Duplicate |
| 31 | Che YanJun et al. (2010) | China | doi:10.3969/j.issn.1004-406X.2010.09.08 | Duplicate |
| 32 | Ruetten et al. (2009) | Germany | doi:10.1097/BSD.0b013e318175ddb4 | Lacking or with incomplete information |
| 33 | Lee et al. (2009) | Korea | doi:10.3340/jkns.2009.46.6.515 | Lacking or with incomplete information |
| 34 | Meng XianZhong et al. (2006) | China | doi: 10.3969/j.issn.1004-406X.2006.09.008 | Wrong outcomes |
| 35 | Anqi Wang et al. (2020) | China | doi:10.2147/TCRM.S283652 | Not Applicable |
| 36 | Yuan Yao et al. (2017) | China | doi:10.1016/j.wneu.2016.11.120 | Not Applicable |
| 37 | Salvatore D’Oria et al. (2023) | Italy | doi:10.5435/JAAOS-D-23-00123 | Not Applicable |
| 38 | Gerald Musa et al. (2024) | Russia | doi:10.1097/MS9.0000000000001600 | Not Applicable |
| 39 | Junlong Wu et al. (2017) | China | doi:10.1016/j.wneu.2017.01.089 | Not Applicable |
| 40 | Chao Liu et al. (2024) | China | doi:10.1016/j.wneu.2016.10.056 | Not Applicable |
| 41 | Ayman A et al. (2013) | Egypt | doi:10.4103/1793-5482.121685 | Not Applicable |
| 42 | Ahmed Zaater et al. (2016) | Egypt | doi:10.1097/WNQ.0000000000000126 | Not Applicable |
| 43 | Erkin Sonmez et al. (2013) | Turkey | doi:10.5137/1019-5149.JTN.7122-12.1 | Not Applicable |
| 44 | Xianglong Zhuo et al. (2009) | China | doi：CNKI:SUN:ZXCW.0.2009-12-007 | Not Applicable |
| 45 | Yongsheng Hu et al. (2023) | China | doi:10.3969/j.issn.1674-3806.2023.11.14 | Not Applicable |
| 46 | Junhai Lu et al. (2022) | China | doi: 10.3969 /j.issn.1001- 5639.2022.02.006 | Not Applicable |
| 47 | Hao Xue (2016) | China | doi：10.3969/j.issn.2095-9958.2016.03-03 | Not Applicable |
| 48 | Xiaogang Hu  (2017) | China | doi：10.3969/j.issn.1005-7234.2017.06.004 | Not Applicable |
| 49 | Tianji Zhang et al. (2017) | China | doi: 10.3969 / j.issn.1674 -3806.2017.07.13 | Not Applicable |
| 50 | Jiancheng Su et al. (2016) | China | doi:10.13795/j.cnki.sgkz.2016.03.018 | Not Applicable |
| 51 | Yinhe Chen et al. (2014) | China | doi:10.13795/j.cnki.sgkz.2014.03.006 | Not Applicable |
| 52 | Guiying Gao et al. (2019) | China | doi:10.3969/j.issn.1008-0287.2019.04.006 | Not Applicable |
| 53 | Liqiang Li et al. (2016) | China | doi:10.1177/0300060516645419 | Not Applicable |
| 54 | Bing Pan et al. (2014) | China | doi:10.3969/j.issn.1003-0034.2014.09.002 | Not Applicable |
