# Supplementary material for: Comparison of 7 surgical interventions for recurrent lumbar disc herniation: A network meta-analysis and systematic review
Source: PLoS One. 2025 Mar 4;20(3):e0309343. doi: 10.1371/journal.pone.0309343 (PMC11878942; doi:10.1371/journal.pone.0309343)
Supplement: S7 Table — (DOCX) [file pone.0309343.s008.docx]

Table 1.GRADE for VAS(back pain)

| Comparison | | | Nature of the evidence | Confidence | Downgrading due to |
| --- | --- | --- | --- | --- | --- |
| PELD | VS | MED | Mixed | Very low | Study limitation;Inconsistency |
|  |  | MIS-TLIF | Mixed | low | Study limitation |
|  |  | TLIF | Indirect | low | Study limitation |
|  |  | Unilat-TLIF | Indirect | low | Study limitation |
|  |  | PLIF | Indirect | low | Study limitation |
|  |  | OD | Mixed | Very low | Study limitation;Inconsistency |
| MED | VS | MIS-TLIF | Mixed | low | Study limitation |
|  |  | TLIF | Indirect | low | Study limitation |
|  |  | Unilat-TLIF | Indirect | low | Study limitation |
|  |  | PLIF | Indirect | low | Study limitation |
|  |  | OD | Indirect | low | Study limitation |
| MIS-TLIF | VS | TLIF | Mixed | Very low | Study limitation;Inconsistency |
|  |  | Unilat-TLIF | Indirect | low | Study limitation |
|  |  | PLIF | Indirect | low | Study limitation |
|  |  | OD | Indirect | low | Study limitation |
| TLIF | VS | Unilat-TLIF | Mixed | low | Study limitation |
|  |  | PLIF | Mixed | Very low | Study limitation;Inconsistency |
|  |  | OD | Mixed | Very low | Study limitation;Inconsistency |
| Unilat-TLIF | VS | PLIF | Indirect | low | Study limitation |
|  |  | OD | Indirect | low | Study limitation |
| PLIF | VS | OD | Mixed | Very low | Study limitation;Inconsistency |

Table 2.GRADE for VAS(leg pain)

| Comparison | | | Nature of the evidence | Confidence | Downgrading due to |
| --- | --- | --- | --- | --- | --- |
| PELD | VS | MED | Mixed | low | Study limitation |
|  |  | MIS-TLIF | Mixed | low | Study limitation |
| MED | VS | MIS-TLIF | Mixed | low | Study limitation |

Table 3.GRADE for ODI(Oswestry disability index)

| Comparison | | | Nature of the evidence | Confidence | Downgrading due to |
| --- | --- | --- | --- | --- | --- |
| PELD | VS | MED | Mixed | low | Study limitation |
|  |  | MIS-TLIF | Mixed | Very low | Study limitation;Inconsistency |
|  |  | TLIF | Indirect | low | Study limitation |
|  |  | PLIF | Indirect | low | Study limitation |
|  |  | OD | Mixed | Very low | Study limitation;Inconsistency |
| MED | VS | MIS-TLIF | Mixed | low | Study limitation |
|  |  | TLIF | Indirect | low | Study limitation |
|  |  | PLIF | Indirect | low | Study limitation |
|  |  | OD | Indirect | low | Study limitation |
| MIS-TLIF | VS | TLIF | Indirect | low | Study limitation |
|  |  | PLIF | Indirect | low | Study limitation |
|  |  | OD | Indirect | low | Study limitation |
| TLIF | VS | PLIF | Mixed | Very low | Study limitation;Inconsistency |
|  |  | OD | Mixed | Very low | Study limitation;Inconsistency |
| PLIF | VS | OD | Mixed | Very low | Study limitation;Inconsistency |

Table 4.GRADE for Complication

| Comparison | | | Nature of the evidence | Confidence | Downgrading due to |
| --- | --- | --- | --- | --- | --- |
| PELD | VS | MED | Mixed | low | Study limitation |
|  |  | MIS-TLIF | Mixed | Very low | Study limitation;Inconsistency |
|  |  | TLIF | Indirect | low | Study limitation |
|  |  | Unilat-TLIF | Indirect | low | Study limitation |
|  |  | PLIF | Indirect | low | Study limitation |
|  |  | OD | Mixed | Very low | Study limitation;Inconsistency |
| MED | VS | MIS-TLIF | Mixed | Very low | Study limitation;Inconsistency |
|  |  | TLIF | Indirect | low | Study limitation |
|  |  | Unilat-TLIF | Indirect | low | Study limitation |
|  |  | PLIF | Mixed | low | Study limitation |
|  |  | OD | Indirect | low | Study limitation |
| MIS-TLIF | VS | TLIF | Mixed | Very low | Study limitation;Inconsistency |
|  |  | Unilat-TLIF | Indirect | low | Study limitation |
|  |  | PLIF | Indirect | low | Study limitation |
|  |  | OD | Indirect | low | Study limitation |
| TLIF | VS | Unilat-TLIF | Mixed | low | Study limitation |
|  |  | PLIF | Mixed | Very low | Study limitation;Inconsistency |
|  |  | OD | Mixed | Very low | Study limitation;Inconsistency |
| Unilat-TLIF | VS | PLIF | Indirect | low | Study limitation |
|  |  | OD | Indirect | low | Study limitation |
| PLIF | VS | OD | Mixed | Very low | Study limitation;Inconsistency |

Table 5.GRADE for Recurrrence

| Comparison | | | Nature of the evidence | Confidence | Downgrading due to | Upgrading due to |
| --- | --- | --- | --- | --- | --- | --- |
| PELD | VS | MED | Mixed | Very low | Study limitation;Inconsistency |  |
|  |  | MIS-TLIF | Mixed | Very low | Study limitation;Inconsistency |  |
|  |  | TLIF | Indirect | low | Study limitation |  |
|  |  | PLIF | Indirect | low | Study limitation |  |
|  |  | OD | Mixed | low | Study limitation |  |
| MED | VS | MIS-TLIF | Mixed | Very low | Study limitation;Inconsistency |  |
|  |  | TLIF | Indirect | low | Study limitation |  |
|  |  | PLIF | Mixed | Very low | Study limitation;Inconsistency |  |
|  |  | OD | Indirect | low | Study limitation |  |
| MIS-TLIF | VS | TLIF | Indirect | low | Study limitation |  |
|  |  | PLIF | Indirect | low | Study limitation |  |
|  |  | OD | Indirect | Moderate | Study limitation | Large effect size |
| TLIF | VS | PLIF | Mixed | Very low | Study limitation;Inconsistency |  |
|  |  | OD | Mixed | low | Study limitation;Inconsistency | Large effect size |
| PLIF | VS | OD | Mixed | low | Study limitation;Inconsistency | Large effect size |
