# Supplementary material for: Comparison of 7 surgical interventions for recurrent lumbar disc herniation: A network meta-analysis and systematic review
Source: PLoS One. 2025 Mar 4;20(3):e0309343. doi: 10.1371/journal.pone.0309343 (PMC11878942; doi:10.1371/journal.pone.0309343)
Supplement: S8 Table — (DOCX) [file pone.0309343.s009.docx]

Table 1.Risk of bias in non-randomised studies of interventions

| Study | D1 | D2 | D3 | D4 | D5 | D6 | D7 | Overall |
| --- | --- | --- | --- | --- | --- | --- | --- | --- |
| Anqi Wang et al.(2020) | Low | Intermediate | Intermediate | High | Low | High | Intermediate | High |
| Yuan Yao et al.(2017) | Low | Intermediate | Intermediate | Intermediate | Low | High | High | High |
| Gerald Musa et al.(2024) | Low | Intermediate | Intermediate | Intermediate | Low | High | Intermediate | High |
| Junlong Wu et al.(2017) | Low | Intermediate | High | Intermediate | Intermediate | High | Intermediate | High |
| Chao Liu et al.(2024) | Low | Intermediate | Intermediate | Intermediate | Low | Intermediate | Intermediate | Intermediate |
| Ahmed Zaater et al.(2016) | Low | Intermediate | Intermediate | Intermediate | Low | Intermediate | Intermediate | Intermediate |
| Erkin Sonmez et al.(2013) | Low | Intermediate | Intermediate | High | Low | High | Intermediate | High |
| Xianglong Zhuo et al.(2009) | Low | High | Intermediate | Intermediate | Intermediate | High | Intermediate | High |
| Yongsheng Hu et al.(2023) | Low | High | Intermediate | High | Low | High | Intermediate | High |
| Junhai Lu et al.(2022) | Low | Intermediate | Intermediate | High | Low | High | High | High |
| Hao Xue(2016) | Low | High | High | Intermediate | Low | Intermediate | High | High |
| Xiaogang Hu (2017) | Low | High | Intermediate | High | Low | High | High | High |
| Tianji Zhang et al.(2017) | Low | High | High | Intermediate | Intermediate | High | Intermediate | High |
| Jiancheng Su et al.(2016) | Low | Intermediate | High | Intermediate | Low | Intermediate | Intermediate | High |
| Yinhe Chen et al.(2014) | Low | High | Intermediate | High | Intermediate | High | Intermediate | High |
| Guiying Gao et al.(2019) | Low | High | Intermediate | Intermediate | Low | High | Intermediate | High |
| Liqiang Li et al.(2016) | Low | Intermediate | Intermediate | High | Intermediate | High | Intermediate | High |
| Bing Pan et al.(2014) | Low | High | Intermediate | Intermediate | Low | High | Intermediate | High |

D1: Bias due to confounding,D2: Bias due to selection of the participants,D3: Bias in classification of intervention,D4: Bias due to deviation from intended

Intervention,D5: Bias due to missing data,D6: Bias in measurements of outcomes,D7: Bias in selection of the reported results

Table 2.Risk of bias in Randomized controlled trial

| Study | D1 | D2 | D3 | D4 | D5 | D6 | D7 |
| --- | --- | --- | --- | --- | --- | --- | --- |
| Salvatore D’Oria et al.(2023) | Unclear | Unclear | Unclear | High | Low | Low | Low |
| Ayman A et al.(2013) | Unclear | Unclear | Unclear | High | Low | Low | Low |

D1:Random sequence generation,D2:Allocation concealment,D3:Blinding of participants and personnel,D4:Blinding ofoutcome

assessment ,D5:Incomplete outcome data,D6:Selective reporting,D7:Other bias
