# Supplementary material for: Comparison of 7 surgical interventions for recurrent lumbar disc herniation: A network meta-analysis and systematic review
Source: PLoS One. 2025 Mar 4;20(3):e0309343. doi: 10.1371/journal.pone.0309343 (PMC11878942; doi:10.1371/journal.pone.0309343)
Supplement: Attachment — Submitted filename: Response to Reviewers.docx [file pone.0309343.s010.docx]

Dear Editor and reviewers

Re: Manuscript ID: PONE-D-24-22399R1 and Title: Comparison of 7 Surgical Interventions for Recurrent Lumbar Disc Herniation: A Network Meta-analysis and Systematic Review.

Thank you for your letter and the reviewers' comments concerning our manuscript entitled “Comparison of 7 Surgical Interventions for Recurrent Lumbar Disc Herniation: A Network Meta-analysis and Systematic Review”(ID: PONE-D-24-22399R1). Those comments are all valuable and very helpful for revising and improving our paper, as well as the important guiding significance to our research, We have studied the comments carefully and have made corrections which we hope meet with approval. The main corrections in the paper and the responses to the editor's and reviewer's comments are as follows:

Responds to the editor's comments:

We are grateful to the reviewers and editorial team for the time and effort they put into evaluating our manuscript. We made detailed revisions to the manuscript as requested. Modify as follows:

1. We have revised the title and figure captions according to the style template of the PLOS ONE manuscript and reflected them in the manuscript.

2. We made changes where there was overlap with the previous publication and this is reflected in the manuscript. (lines 66-70)

3. We have sorted out the raw data needed for this study and uploaded it as supporting information. At the same time, we have attached the description of supporting information files at the end of the manuscript.( line 158 and lines 379-398)

4. We have uploaded the full PRISMA checklist as support information.

Responds to the reviewer's comments:

Reviewer #1: We sincerely thank you for your comments and suggestions, which have greatly facilitated our research.

Reviewer #2:

1. Response to comment:( **It was not searched systematically according to the principles of PICO, the MeSH were not clear, and the free words were not comprehensive.**)

Response:

We think this is an excellent comment. We possess a profound comprehension of the principles of PICO significance in clinical research, and the meticulous exploration of pertinent literature is paramount. Consequently, we have refined the MeSH and free words of this study, conducted additional searches for related studies, and integrated the revised MeSH and free words into the manuscript.(lines 99-109)

2. Response to comment:(**The retrieval database is not comprehensive, and the databases that must be retrieved for meta analysis: pubmed, embase, cochrane library; Database to be retrieved as far as possible: web of science, clinicalrials gov,Google Scholar.**)

Response:

We appreciate your valuable comments. Yes, a comprehensive search of articles from different databases is essential, so we researched relevant articles in different databases again, for example, PubMed, Embase, MEDLINE, Cochrane Library, Web of Science, Google Scholar and China National Knowledge Infrastructure databases, meanwhile, the results of the search are integrated into the manuscript. (lines 97-99 and Fig.1)

3. Response to comment:( **the registration of the plan had not been completed in PROSPERO.**)

Response:

Thank you very much for your comments. the registration of the study had been completed in PROSPERO (Prospero registration number: CRD42024545798). (lines 113-115)

4. Response to comment:( **The studies included in the meta-analysis should follow the principle of homogeneity, and the combination of RCT and retrospective study can only increase the heterogeneity. It is recommended that RCT and retrospective study be subgrouped.**)

Response:

We appreciate your valuable comments. Yes, In the meta-analysis, the included study should follow the principle of homogeneity in order to improve the credibility of the evidence, which we deeply agree with. At the same time, the subgroup analysis of RCT and retrospective studies will help to further improve the credibility of the conclusions. We sincerely thank you for your suggestion. In this study, we are also aware of this, but considering that in the clinical study of orthopedic surgery, The RCT is challenging because the choice of surgical methods will be affected by the choice of patients, although some studies have described randomized controlled trials. At the same time, in the included study, the number and sample size of randomized controlled trials are relatively small, which is also one of the limitations of this study. If we analyze the randomized controlled trials in groups, there is a risk of chance. Therefore, after a detailed discussion, we combined randomized controlled trials with a retrospective study in this study. Meanwhile considering the heterogeneity of the study, we adopt the random effect model to analyze and draw a conclusion.

We deeply recognize the limitations and shortcomings of this study, so in future research, we will include more high-quality, low-risk randomized controlled trials for analysis, increase the sample size, reduce the risk of heterogeneity, and improve the shortcomings of the study. Thank you for your valuable advice again.

As researchers, we deeply recognize the importance of the review comments. We tried our best to improve the manuscript which will not influence the content and framework of the paper. We appreciate for editors' and reviewers’ warm work earnestly and hope the correction will meet with approval. Once again, thank you very much for your comments and suggestions.

Best regards!
